# Supplementary material for: Resolving the full spectrum of human genome variation using Linked-Reads
Source: Genome Res. 2019 Apr;29(4):635–45. doi: 10.1101/gr.234443.118 (PMC6442396; doi:10.1101/gr.234443.118)
Supplement: Supplemental Material [file supp_gr.234443.118_Supplemental_longranger-2.2.zip › longranger-2.2.tar.bz2!/lr2.2-public/lib/stan/cmdstan/stan/doc/api/html/namespacestan_1_1common_1_1recorder.html]

Stan: stan::common::recorder Namespace Reference


|  |  |
| --- | --- |
|  | Stan  2.5.0  probability, sampling & optimization |


- Main Page
- Related Pages
- Namespaces
- Classes
- Files

- Namespace List
- Namespace Members

All Classes Namespaces Files Functions Variables Typedefs Enumerations Enumerator Friends Macros Pages

- stan
- common
- recorder

Classes

stan::common::recorder Namespace Reference

|  |  |
| --- | --- |
| Classes | |
| class | csv |
|  | Writes out a vector as string. More... |
|  | |
| class | filtered\_values |
|  | |
| class | messages |
|  | |
| class | no\_op |
|  | Does nothing. More... |
|  | |
| class | sum\_values |
|  | |
| class | values |
|  | |


---

[ Stan Home Page ]

*© 2011–2014,
Stan Development Team.*
